# Perceptions of preparedness for the first medical clerkship: a systematic review and synthesis

**DOI:** 10.1186/s12909-016-0615-3

**Published:** 2016-03-12

**Authors:** Laura Surmon, Andrea Bialocerkowski, Wendy Hu

**Affiliations:** Medical Education Unit, School of Medicine, Western Sydney University, Bdg 30, Campbelltown Campus, Locked Bag 1797, Penrith South DC, NSW 1797 Australia; School of Allied Health Sciences, Griffith University, Gold Coast, QLD Australia

**Keywords:** Medical school, Clerkship, Transition, Preparedness, Clinical education, Experiential learning, Workplace based learning, Informal learning

## Abstract

**Background:**

The transition from university-based to clerkship-based education can be challenging. Medical schools have introduced strategies to ease the transition, but there has been no systematic review synthesizing the evidence on the perceptions of preparedness of medical students for their first clerkship to support these interventions. This study therefore aimed to (1) identify and synthesize the published evidence on medical students’ perceptions of preparedness for their first clerkship, and (2) identify factors that may impact on preparedness for clerkship, to better inform interventions aimed at easing this transition.

**Methods:**

Electronic databases (Medline, Journals@Ovid, CINAHL, ERIC, Web of Science, Embase) were searched without restriction and secondary searching of reference lists of included studies was also conducted. Included studies used quantitative or qualitative methodologies, involved medical students and addressed student/supervisor perceptions of preparedness for first clerkship. The first clerkship was defined as the first truly immersive educational experience during which the majority of learning was vocational and self-directed, as per the MeSH term ‘clinical clerkship’ and associated definition. Using an inductive thematic synthesis approach, 2 researchers independently extracted data, coded text (from results and discussion sections), and identified themes related to preparedness. Any disagreements were resolved by discussion and findings were then narratively synthesized.

**Results:**

The initial search identified 1214 papers. After removing duplicates and assessing abstracts and full articles against the inclusion criteria, 8 articles were included in the review. In general, the body of evidence was of sound methodological quality. Ten themes relating to perceptions of preparedness of medical students for their first clerkship were identified; competence, disconnection, links to the future, uncertainty, part of the team, time/workload, adjustment, curriculum, prior life experiences and learning.

**Conclusions:**

Eight of the ten themes related to perceptions of preparedness are potentially amenable to curricula strategies to improve the transition experience. The evidence supports clinical skills refreshers, clarification of roles and expectations, demystification of healthcare hierarchy and assessment processes and student-student handovers. Evidence also supports preclinical educational strategies such as enhancing content contextualization, further opportunities for the application of knowledge and skills, and constructive alignment of assessment tasks and pedagogical aims.

**Electronic supplementary material:**

The online version of this article (doi:10.1186/s12909-016-0615-3) contains supplementary material, which is available to authorized users.

## Background

The inevitable transition from preclinical, university-based learning and teaching to clinical learning, which is undertaken in health facilities (i.e. a clerkship), is one of several important transitions during medical training. Evidence suggests that this transition is a source of high levels of stress and anxiety for medical students [1, 2]. This is of concern, as stress can impact on cognitive function and learning [3]. A potential source of this stress and anxiety may relate to students’ perceptions of being inadequately prepared for clerkship. Therefore, improving medical students’ perceptions of preparedness for their first clerkship may ease the transition while facilitating learning within the clinical setting.

The academic progression of medical students in the years preceding clerkship is often based on performance in nonclinical assessment tasks. These assessment tasks generally focus on knowledge of the basic sciences and basic clinical skills. Basic clinical skills may be taught in simulated and/or actual healthcare settings, depending on the pedagogical approach underpinning the curriculum [4]. It is evident that additional factors could influence student preparedness to enter the clinical learning environment, including individual student characteristics, the nature of university-based pedagogy and the (lack of) structure in the clinical environment into which students enter [5]. By better understanding these factors, educational activities may be developed to better prepare medical students for this important transition. By improving students’ transition experience, well-designed educational interventions could minimize any negative impacts on student learning or wellbeing during this period [6].

Many medical schools have identified the need to improve preparedness and transition into the clinical learning environment [7, 8]. This has seen the introduction of longitudinal programs that scaffold communication and basic clinical skills throughout the university-based curriculum, with some medical schools implementing short transition courses or pre-clerkships [7, 8]. While these interventions seemed intuitively sound, no systematically collected and synthesized evidence exists regarding the effect of these interventions on perceptions of preparedness of medical students for their first clerkship. This evidence is required to provide a sound justification for implementing educational interventions targeted at addressing student preparedness, and to inform the development of new, evidence-based strategies that address significant factors known to influence preparedness [5].

Therefore, the aim of this systematic review was to rigorously: (1) identify and synthesize the published evidence on medical students’ perceptions of preparedness for their first clerkship, and (2) identify factors, through thematic analysis, that may impact, either negatively or positively, on preparedness for clerkship, to inform medical educators and curriculum designers.

## Methods

The systematic review methodology was informed by the 2009 PRISMA Statement on the content and reporting of systematic reviews [9] (Additional file 1: PRISMA Checklist) and in line with the enhancing the transparency in reporting the synthesis of qualitative research (ENTREQ) statement [10]. Systematic review and synthesis of primary and secondary data employs the same methodology used in qualitative data analysis to analyze articles that meet the inclusion criteria for the systematic review [10, 11].

### Data sources and search strategy

A comprehensive search of seven electronic databases (Medline, Journals@Ovid, CINAHL, ERIC, Web of Science, Embase and PsycINFO) was conducted. The search strategy was based on search strategies utilized in published systematic reviews on clinical education [12–14]. Synonyms and Medical Subject Heading [MeSH] terms for “clinical placement”, “supervisor”, “medical students”, “discipline of medicine”, “first exposure”, “preparedness” were combined using OR and AND where appropriate, as illustrated in Additional file 2: Table S1. The same search strategy was used for all electronic databases except that MeSH terms were used as Abstract/Title searches where appropriate, or excluded when a database did not support the MeSH field searches. All electronic databases were searched from the date of database inception with no limits on language or year of publication. Secondary searching of the reference lists of included articles was also conducted to identify additional primary evidence. The full search strategy used for MeSH databases is shown in Additional file 2: Table S1.

### Study eligibility and selection

The titles and abstracts of all citations identified via the database search and secondary searching were evaluated for inclusion into this study using the screening question ‘Does the article appear to address the first clerkship in medical education?’. The first clerkship was deemed to be the first truly immersive educational experience during which the majority of learning was vocational and self-directed, as per the MeSH term ‘clinical clerkship’ and associated definition [15]. Duplicates were subsequently removed. Abstracts, followed by full-text articles, were then reviewed against the following inclusion criteria:

1. Be published in full in English and contain data from primary studies
2. Used any type of quantitative or qualitative methodology, to gain multiple perspectives of preparedness
3. Involved medical students in graduate or undergraduate professional entry-level medical programs
4. Addressed the perceptions of preparedness of medical students for their first clinical placement, from the perspectives of medical students and/or their supervisors

One researcher (LS) screened all citations. The abstracts and full text versions of those citations that addressed the screening question were independently reviewed against the selection criteria by two researchers (LS and AB). Reference lists of the articles, which met the inclusion criteria, were subsequently screened and evaluated against the inclusion criteria. The list of included articles was then compared between researchers. When disagreements occurred, consensus was reached by repeated discussion and resolution of the different interpretations.

### Data extraction

Methodological rigour of each included study was then evaluated. The method of evaluation was dependent on the study design. Quantitative studies which collected data via a questionnaire were evaluated using the comprehensiveness of reporting criteria [16] whereas studies which used a qualitative design were evaluated using the Critical Appraisal Skills Program (CASP) checklist for qualitative research [17]. Two researchers (LS and AB) independently evaluated each primary study. Any disagreements were resolved by discussion.

The researchers then extracted the following data from each primary study, using a purpose-built, standardized data extraction tool:

- study context, including the country of origin, length and type of entry level degree, the characteristics of the first clinical placement
- aims of the study
- participant characteristics, including the number and gender, and whether the participants were students or supervisors
- the results with respect to student preparedness and the perspective from which this was evaluated (i.e. the student or the supervisor)

Two researchers (LS and AB) independently performed this process. Disagreements were again resolved by discussion.

### Data syntheses and analyses

Agreement between the two researchers on the methodological quality of the included studies was established by calculation of percentage agreement and the Kappa statistic.

In addition, using an inductive approach, all text from the results and discussion sections of each article were imported into a qualitative data analysis software program, HyperResearch [ResearchWare, Randolph, Massachussets, USA], for coding and thematic synthesis [11]. Two researchers (LS and WH) independently performed line-by-line free coding of text that related to medical students’ or their supervisors’ perceptions of students’ preparedness for their first clerkship. Coded text included primary evidence in the form of original quotations, and secondary evidence included results summaries and interpretations of results by the authors of each study. Evidence from questionnaire items and associated data were coded in the same manner. Each subsequent article was analyzed by adding coded text to existing themes or by creating new themes when required. The two researchers (LS and WH) then discussed the independently identified themes to discover conceptually equivalent, or unique themes. Themes were amalgamated and condensed where appropriate until a final set of themes and their definitions were agreed upon. Using an integrative approach, themes and extracted data were then synthesized narratively, using a thematic synthesis approach, to address each aim of the study [18, 19].

## Results

### Trial flow

**Fig. 1:**
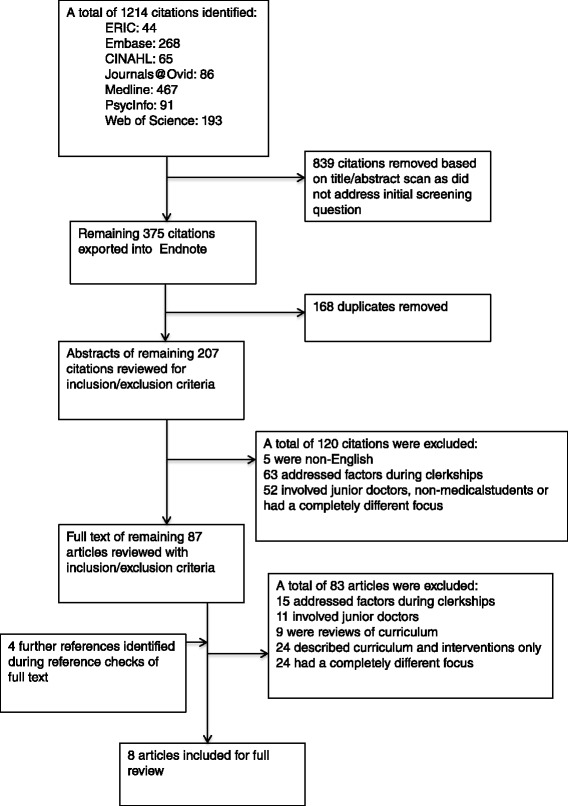
The process used to select the appropriate studies for inclusion

A total of 1,214 articles were identified using the search strategy. Of those, 375 met the screening question. The 168 duplicates were removed, leaving 207 articles for evaluation against the selection criteria. One hundred and twenty articles were excluded based on review of their abstract. An additional four articles were identified based on secondary searching of reference lists. A total of 83 articles were then excluded after reviewing their full text version against the inclusion criteria. Thus a total of 8 articles published between 2000 and 2010 were included in this systematic review [20–27] (Fig. 1). Three studies used a quantitative method to administer a questionnaire, [20, 21, 23] whereas the remaining five studies were qualitative in nature [22, 24–27].

### Methodological rigour

Of the three studies [20, 21, 23] that used a quantitative survey, 100 % agreement (K = 1.0) was gained with respect to the comprehensiveness of reporting. These studies were generally considered of sound methodological rigour, reporting the response rate, characteristics of participants in their sample, and descriptions of the survey instruments and statistical analyses (Additional file 3: Table S2). However, none of the studies reported piloting the survey instruments, one failed to describe survey development [20] and only one of the two studies that included open-ended questions [20] provided participant quotations and details of the qualitative analysis.

Complete agreement was also reached between the researchers with respect to the methodological rigor of the five qualitative studies (100 % agreement, K = 1.0) [22, 24–27]. All of the five studies met all of the 10 criteria in the CASP checklist for qualitative research.

### Study characteristics

Of the eight articles included in this systematic review, two were conducted in the United Kingdom, three in the United States of America and three in the Netherlands. All were published between 2000 and 2010. The settings for six of the studies varied from 4-year graduate programs [23, 25] to 5 or 6-year undergraduate programs [20–22, 26]. Four programs used a problem based learning (PBL) preclinical curriculum [20–22, 26] and two used a more traditional curriculum design [23, 25]. One study did not specify the type of curriculum used [27] while another study analyzed data obtained from 10 North American medical schools but did not provide details on the curricula of any of the schools [24].

The aims of each study varied, however all were related to seeking data on the experiences or perceptions of medical students and/or clerkship staff on the transition to clerkship, differences between preclinical and clinical education and preparation/preparedness for clerkship. Five studies employed purely qualitative methodology, [22, 24–27] two used surveys with open-ended questions [20, 21] and one used a survey, which contained only closed-ended questions [23]. All studies collected data from medical students. In three studies, data were also collected from clerkship staff (Additional file 4: Table S3).

## Results

Ten themes related to perceptions of preparedness for first clerkship emerged from the analysis. These themes were grouped into domains based on type of influence and modifiability. The supplementary material provides illustrative examples of coded text (Additional file 5: Table S4) and an unabridged discussion on each theme.

### Potentially modifiable through curriculum

i. Positive and negative influences
  Competence
    Seven of the eight studies provided evidence to support the theme of competence [20–24, 26, 27]. At the beginning of clerkships, students expressed concern over perceived deficiencies in knowledge and/or skills, which they perceive as a cause of stress. The reason for such deficits, according to students, was the decontextualized and non-integrated nature of their preclinical studies [24].
    Students were often concerned about appearing foolish in front of patients or supervisors [26, 27], which may be related to the finding that in terms of clinical skills, the expectations of medical students and preclinical staff were significantly higher than those of clerkship staff [23]. There were, however, students who felt satisfied with their level of skills and knowledge and/or who accepted their feelings of being deficient in knowledge and skill as inevitable, understanding that they were undertaking clerkships to learn [20, 21, 26].
  Curriculum
    All studies reported that the curriculum informed preparedness for transition, and references to actual or suggested educational strategies to increase preparedness for clerkship are included in this theme. The most frequently mentioned strategy was the clerkship orientation. These were designed to assist students to integrate into the clinical environment and to refresh their knowledge and skills. Students found non-didactic introductions improved their preparedness but felt that clerkship introduction activities should address roles and expectations, hospital hierarchy, time management and self-care, and assessment [21, 22, 24, 25]. The value of the insights of senior students was frequently mentioned, either their involvement in preclinical curriculum [20, 25] or to provide advice about their experience to the incoming students at the beginning of each rotation [25].
    In terms of preclinical curriculum, students saw the benefit of clinical skills programs such as weekly bedside teaching, longitudinal mentoring, early contact with patients and problem-based or case-based learning in improving preparedness for the first clerkship. For such educational strategies to be effective however, activities needed to be authentic as opposed to tokenistic or simulated, although as a starting point, students did value simulated patient encounters and skills lab activities. In hindsight, students reported valuing more contextualized and clinically integrated preclinical curriculum with more opportunities to apply what they were learning. Likewise, preclinical assessment, which was designed to test and therefore encourage recall rather than understanding, was also recognized by students as a barrier, or at least unhelpful to adapting to the clinical learning environment [21]. Further, a gradual transition was preferred In terms of shifting to learning in the clinical environment [20, 21, 26, 27].
  Learning
    Six studies contributed evidence to this theme [20–22, 24, 26, 27]. Differences in learning styles and in the learning environment, including the reframing of patients as tools for learning, influenced students’ preparedness. Learning often needed to be at a faster pace and to a greater depth during clerkship and students frequently reported difficulties associated with switching from learning passively through lectures and textbooks in the preclinical curriculum to learning from patients and people in a clerkship [21, 22, 24, 26, 27]. Students reported difficulty applying their knowledge and skills, likely owing to the limited opportunity to apply what they had learnt in the preclinical years [20–22, 24, 26].
    Clerkship staff referred to the switch in learning styles and related it to the structure of assessment in preclinical education. This encouraged students to learn for assessment [24, 27] and therefore did not prepare them for the learning required in the clerkships, which was continuous and self-directed and relied on students’ abilities to self-reflect, identify and search out information to fill gaps in their knowledge [24].
    These findings were not only restricted to students who were studying in the traditional non-PBL programs with little preclinical practice experience but also to newer models with early clinical experiences [21, 22, 26, 27]. Not all students in each program struggled with the new style of clinical learning required; statements relating to an increase in motivation, deeper learning and retention and more enjoyment of the learning process were common [20, 21, 26, 27].
ii. Negative influences
  Disconnection
    Four of the eight studies provided evidence to support the theme of disconnection [20, 22, 24, 27]. Many students seem to commence their first clerkship lacking an understanding of the trajectory of learning and development from medical student to graduate. This affected their ability to draw on and apply knowledge and skills and thus they felt ill-prepared, even if they had indeed acquired the specific knowledge and skills required for clerkship. Contributing to this was a sense of disconnection experienced between learning in the preclinical and clinical years [20, 22, 24, 27]. Some students came late to the realization that the preclinical curriculum was designed to prepare them for the clinical years [20, 22]. In addition, students felt that they were ill prepared by the lack of opportunity to apply their learning in the preclinical years. They regarded this as demotivating and contributing to their failure to see the relevance or connection between theory and practice [24, 27]. Clerkship staff also perceived the disconnection between the preclinical and clinical curriculum to be a contributing factor for ill preparedness and difficulties in transitioning to clinical learning [24].
  Uncertainty
    A prominent theme present in all except one study was the uncertainty and insecurity students experienced during their first clerkship in terms of what was expected of them as clerks performing actual clinical practice [20–22, 24–27]. Students frequently mentioned not knowing the unwritten rules [20–22, 24–27]. Uncertainty about how to adjust their learning style to a new setting where the priority was clinical service delivery and not necessarily learning, and how to apply and integrate their preclinical, theoretical knowledge were also common [21, 22, 24–26]. The structure and processes for assessment and feedback were often unclear and mystifying to clerkship students at the beginning of their rotation [24]. Variations in approach to patient care and other aspects of clinical practice from their supervisors and other clinical staff were also reported to contribute to uncertainty [24, 26]. Likewise, the considerable differences in styles and approaches of different clinical educators required students to adapt frequently [24]. Clerkship staff often failed to appreciate the impact of such factors on student learning and ease of transition [24].
  Part of the Team
    Professional socialization or being part of a team was reported in seven of the eight studies [20–22, 24–27]. Students were often unsure of the health service hierarchy and how different professions work together [27] as well as how to behave or act [20, 22, 24–26]. Such preoccupations were reported to have affected their learning in these early weeks but tended to reduce as they became more comfortable in their new environment. There was a general feeling that clearer guidance on the role of a clerk, hospital hierarchy and boundaries would have assisted with the transition to the clinical environment.

### Personal characteristics

i. Positive influences
  Prior Life Experiences
    Five studies provided evidence contributing to this theme [22, 24, 26, 27].
    There was evidence that maturity and life experiences before or during medical school could impact on preparedness for students’ first clerkship. Students who had had more experience with patients, or in more varied settings prior to medical school or during their preclinical education, felt more prepared for their first clerkship [20, 22]. Such students also seemed more able to articulate and draw on their preclinical education in the transition to clerkship, thus increasing their perceptions of preparedness [20].

### Adaptation and transition

#### Links to the future

Two of the eight studies provided evidence to support the theme of links to the future [26, 27]. During the initial period of their first clerkship experience, students reported the need to frequently take stock of what it means to be a clerk and how this role fits with becoming a doctor [26]. This connection signaled an important stage in the development of a medical professional identity. Contact with patients and interactions with doctors and other members of the healthcare team were now a motivation to learn; students could begin to put everything into perspective, grow a sense of identity and feel psychologically closer to their final goal of being a doctor [26, 27]. In contrast to transitioning from the preclinical years, developing a sense of connection to their future vocation during their first clerkship did not seem to cause concern about preparedness for graduate practice. On the contrary, it seemed to be a highly motivating part of the transition.

#### Time/workload

Time or workload was reported in six of the eight studies [20–22, 24–26]. Students entering the clinical learning environment had to learn how to balance time on the wards with study, social and personal obligations and travel. Many students felt that they had insufficient time for studying. As well as longer hours, the pace and intensity of the experience, with many patients to see in a short time, was new. This resulted in difficulties absorbing information and spending sufficient time with individual patients to learn [26]. The experience of an abrupt change in workload intensity left students feeling mentally and physically exhausted. However, some students reported being prepared for this change and accepted it as part of training to become a doctor [26, 27].

#### Adjustment

Adjustment was reported in five of the eight studies [20, 22, 24, 26, 27]. It encompassed the adjustment to the clinical environment, student attitudes towards the period of adjustment, as well as the shock or ease of transition [20, 22, 24, 26, 27]. Students felt that the transition to clerkship and clinical learning was too abrupt. Of concern, a few students reported considering leaving medical school at the start of clerkship [21]. Suggestions that a more gradual progression to full time clerkship might reduce student stress or better prepare students were common [20, 27]. There were also suggestions for other ways to ease the transition, such as following a patient through a positive experience such as a pregnancy, [27] and increasing experience in practice settings during the preclinical years [20]. Adjustment involved building the confidence to; talk to patients; perform clinical tasks; ask questions of their supervisors; and interact with other members of the healthcare team. Students needed to understand their roles and what was expected of them; to this end adjustment was easier in more structured clerkships, with set tasks and set time-frames [26]. There was also a need to adjust to a new style of learning and a workload that left little time for other activities. During their first clerkships students experienced an increased sense of responsibility to patients. Encountering serious illness, distress and death meant being confronted by their own often intense emotional reactions [24, 26, 27]. This, coupled with the drive to learn, albeit in a completely different way, contributed to the mental, emotional and physically draining nature of the adjustment period.

Mature students appeared to adjust more easily than school-leaver/younger students to clerkship. However, it seemed to be prior life experiences rather than age itself that was the underlying factor. Students in different studies acknowledged and accepted the inevitability of a period, or several periods, of difficult transitions during medical training [20, 24, 26]. Overwhelmingly, although the aforementioned factors led to a certain degree of shock in transition, overall students found that the move to the clinical environment was exciting, and before long it stimulated their learning. However, students also strongly expressed the need for a more thorough introduction to the clerkship experience to better prepare them for the transition.

In summary, factors during the first clerkship that could impact on student learning were uncertainty about roles and expectations including boundaries, the amount and context of prior experiences with patients, workload and time for study, confidence and the different styles and approaches of clinical teaching staff.

## Discussion

**Fig. 2:**
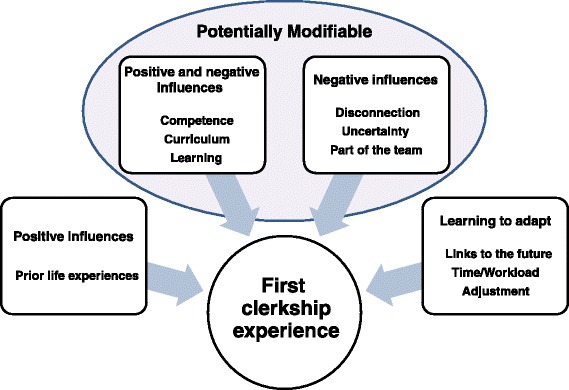
Themes illustrating factors that affect the perceptions of preparedness for the first clinical clerkship or adjustment processes to be learnt

This is the first systematic review to synthesize quantitative and qualitative evidence on medical student perceptions of preparedness for their first clerkship. The results indicated that there are 10 key themes relating to perceptions of preparedness for their first clerkship, which are visually represented in Fig. 2. These themes provide a starting point with which to examine current curricula and develop targeted educational strategies to increase students’ perceptions of preparedness for the first clerkship.

The majority of themes affecting perceptions of preparedness are potentially modifiable through curricula strategies (COMPETENCE, CURRICULUM, LEARNING, DISCONNECTION, UNCERTAINTY, PART OF THE TEAM) whereas the theme PRIOR LIFE EXPERIENCES is a personal characteristic of individual students. The factors existing within the themes of TIME/WORKLOAD, LINKS TO THE FUTURE and ADJUSTMENT, such as the increase in workload, the accelerated development of a professional identity and the adjustment period during clerkship, did not impact directly on perceptions of preparedness but were contributory factors to the disruptive experience of transition.

As illustrated by Fig. 2, some themes could impact positively or negatively on perceptions of preparedness (COMPETENCE, CURRICULUM and LEARNING) while others were largely negative influences (DISCONNECTION, UNCERTAINTY and PART OF THE TEAM). Prior life experiences and maturity only contributed positively to perceptions of preparedness in PRIOR LIFE EXPERIENCES. The themes of LINKS TO THE FUTURE, TIME/WORKLOAD and ADJUSTMENT however, were different in that they addressed the inevitability and necessity of adapting to learning in the clinical environment.

Importantly, there is likely a difference between *feeling* ill prepared and actually *being* ill prepared by having insufficient skills and knowledge. For instance, students frequently reported that they felt they had deficiencies in knowledge of anatomy and pharmacology, and interpretation of investigations, but it is unclear whether these were actually present or were significant to require alterations to the preclinical curriculum. It is also not known whether the perceived deficiencies arose from a failure to manage the expectations of students and supervisors. Supporting this, not all students from the same study were unaccepting of their deficiencies, acknowledging that they were clerks in order to learn [20, 26]. Preclinical and clinical staff did not always agree on the level of knowledge and skills required for clerkship [23]. It was then unsurprising that students were unclear on the knowledge and skills that were necessary prerequisites for clerkship, adding to student anxiety about the adequacy of their preparations.

The uncertainty around not just knowledge but also roles, appropriate behavior, assessment and boundaries provides evidence for more clearly communicating performance expectations. Inevitably there will be differences between different clerkships sites in teaching styles, learning environment, team relationships and processes, so the passing on of a “survival guide” from previously placed students may assist the adjustment of new students. Other ways of addressing this uncertainty may be through detailed job descriptions or duties statements or by increasing the accessibility of supervisors and staff with scheduled regular debriefs and meetings. These would also create more structure within clerkships.

The sense of disconnection between the preclinical and clinical years and some of the difficulties faced during adjustment such as switching to learning from patients and people, and experiencing illness, distress and death, provide evidence for graduated or staged experiential experiences in preclinical years. Opportunities for reflection, sensitive discussion and timely exploration of student experiences and its implications for their journey to becoming a doctor should be offered to extract value from clinical learning. For effective learning, it is suggested that such experiences be openly and explicitly examined, such as in small group discussions and mentoring sessions. This conclusion is consistent with the principles of Kolb’s Learning Cycle, which has recently been applied to residency education [28].

Despite the integrative aims of PBL [29], students from PBL-based programs still reported needing to change from learning backwards from diagnosis to symptoms, to reasoning forwards from patient symptoms to diagnoses. A PBL curriculum was still associated with a perceived disconnection between learning in the preclinical and clinical years due to limited integration of basic and clinical knowledge [22]. One reason may be the structure of assessments in the preclinical years, which may not be pedagogically aligned with PBL, and the tendency of students to learn only for their next assessment task. There was evidence that greater emphasis should be placed on contextualization in the preclinical curriculum to facilitate deeper learning and assist students to see the connection to learning in the clinical years. Curriculum that embraces contextual learning, stemming from a constructivist approach, acknowledges that student learning is anchored within the context of each situation [5]. Ways in which this might be achieved include introducing more opportunities to apply knowledge in the preclinical years, providing clinical shadowing opportunities, utilizing clinical cases to teach basic science, and demonstrations of the clinical reasoning process in action. Importantly though, is the need to ensure that the program of assessment encourages deeper learning and reflects the pedagogical aims of the teaching activities.

The themes of TIME/WORKLOAD, PART OF THE TEAM and ADJUSTMENT contained factors that were not directly related to perceptions of preparedness but provided evidence for addressing certain factors in preclinical or transitional curricula. An example was students’ intensive efforts to find their place on the healthcare team and to understand the roles of others during the first few weeks of their first clerkship. This ‘steep learning’ can be expected as a normal part of the transition to any healthcare workplace [30]. However, to reduce the surprise and shock felt by some students and its subsequent stress and disruption on learning, students could begin their socialization earlier through early supported experiences in the setting, for example via shadowing, mentoring or brief preclinical clerkships. Alternatively, an accepted framework for learning about, and working with, health care teams is interprofessional education (IPE) [31, 32]. However, while IPE may assist students to adjust, a recent systematic review was unable to draw conclusions on the effectiveness of IPE in terms of patient, practitioner or collaborative outcomes [33].

That many students struggled with the increase in workload and demands on time indicates that students entering their first clerkship might benefit from effective time management training. Reminders that this includes making time for self-care could be highly relevant given the evidence that this transition is a source of high levels of stress and anxiety [1, 2]. Moderating expectations to allow for time to adjust to new levels of responsibility, build confidence, confront their new attitudes, values and emotions, and adapt to a new learning style may also ease the transition experience.

The strength of this systematic review and confidence in the findings arises from the methodological rigor of the included studies, which was generally high, and the quality of the review protocol which; utilized a comprehensive range of databases to ensure no relevant primary studies were omitted; combined qualitative and quantitative data; utilized thematic and quantitative analyses; ensured independence and agreement between reviewers; and met PRISMA guidelines.

There are however, several limitations including the small number of studies included. Of those studies, most were conducted at a single institution. As different studies had different aims, data relevant to this review may have been collected, but not reported. The comprehensiveness of systematic reviews of qualitative evidence also relies on the accessibility of primary data in publications for secondary coding [10]. Only one study, Prince et.al. [22], compared participants and non-participants by analyzing their academic performance and finding insignificant differences. It is possible, and highly likely for most studies, that the participants were a highly selected sample of students and teachers. Volunteer participants may tend to be outliers with greater anxieties and expectations of achievement or those who have had bad experiences and wish to voice them. However, it appears that there is little reported research data on this key transition experience, even when a systematic and comprehensive search strategy was applied to locate evidence.

This review reported on the views of students who have already commenced their clinical clerkship, and excluded studies that sampled pre-clinical student perceptions of preparedness. However, by focusing on students who had actually experienced this transition, the findings are more likely to accurately capture the experience. While this may introduce recall bias, [34] the opportunity to reflect on the experience may also yield greater insights on what curricular strategies are effective for reducing the disruptive stress of transition.

In terms of future research, interventional studies utilizing some of the aforementioned curricular recommendations and assessing outcomes other than student and/or supervisor perceptions would be invaluable. However there are obvious difficulties in proving causal effects with even the most rigorously designed studies in this complex space of learning. Further, it may be useful to conduct research on the qualities or conditions under which students do not perceive this transition to be stressful and whether this is a functional response signaling successful adaptation and socialization, or a dysfunctional response signaling a failure of insight and substandard clinical abilities.

### Implications

Taken as a whole, the evidence presented in this systematic review is in favor of strategies that could be implemented at the beginning of or immediately prior to clerkship. Based on the evidence, such strategies should include clinical skills refresher courses, clarification of roles and expectations, demystification of the healthcare hierarchy and assessment processes and the involvement of more senior clerkship students in student-student handovers. In addition, preclinical educational interventions could include; authentic learning experiences with patients, which is also likely to increase motivation; enhancing the contextualization of content; and providing more opportunities for application of knowledge and skills. Other options include shadowing or clinical mentoring and better alignment of assessment tasks with the pedagogical aim of integrating basic with clinical science in medical curricula.

## Conclusions

This is the first systematic review to synthesize quantitative and qualitative evidence regarding factors affecting the perceptions of medical students’ preparedness for their first clerkship. It provides empirical evidence for particular curricular strategies to improve the transition experience. Based on data from eight studies, which included a total of 628 students and 152 clerkship staff, 10 themes underpinning preparedness of the first clinical clerkship were identified, 6 of which are potentially modifiable through curricula strategies.

The findings can be used to inform medical educators and curriculum designers in the development of new, or strengthening of existing, evidence-based educational strategies, to increase medical students’ perceptions of preparedness for their first clerkship. By addressing underlying causes for student stress and anxiety during transition, negative effects on learning may be more effectively ameliorated, so that students are better placed to optimize their learning during this important time.

## Ethical approval

Not applicable.

## Additional files

Additional file 1: PRISMA Checklist. (DOC 63 kb) Additional file 2: Table S1. Search Strategy. (DOCX 31 kb) Additional file 3: Table S2. Comprehensiveness of reporting—survey studies (DOCX 36 kb) Additional file 4: Table S3. Characteristics of Included Studies (DOCX 45 kb) Additional file 5: Table S4. Themes, theme descriptions and examples of coded text (DOCX 94 kb)
