# Supplementary material for: Perceptions of preparedness for the first medical clerkship: a systematic review and synthesis
Source: BMC Med Educ. 2016 Mar 12;16:89. doi: 10.1186/s12909-016-0615-3 (PMC4788861; doi:10.1186/s12909-016-0615-3)
Supplement: Additional file 2: Table S1. — Search Strategy. (DOCX 31 kb) [file 12909_2016_615_MOESM2_ESM.docx]

**Additional file 2: Table S1**

|  | Concept | Term | Field |
| --- | --- | --- | --- |
| 1 | Clinical placement | “clinical placement*” | [Title/Abstract] |
| 2 |  | clinical clerkship | [MeSH Terms]^a^ |
| 3 |  | “student placement*” | [Title/Abstract] |
| 4 |  | “clinical education” | Title/Abstract |
| 5 |  | “clinical experience*” | Title/Abstract |
| 6 |  | “field work” | [Title/Abstract] |
| 7 |  | immersive | [Title/Abstract] |
| 8 |  | preceptor* | [Title/Abstract] |
| 9 |  | “student practicum*” | [Title/Abstract] |
| 10 |  | placement* | [Title/Abstract] |
| 11 |  | “practice experience*” | [Title/Abstract] |
| 12 |  | “clinical attachment* “ | [Title/Abstract] |
| 13 |  | “experiential learn*” | [Title/Abstract] |
| 14 |  | "practice based" | Ttitle/Abstract] |
| 15 |  | family practice, education | [MeSH Terms]^b^ |
| 16 |  | "workplace based" | [Title/Abstract]) |
| 17 |  | #1 OR #2 OR #3 OR #4 OR #5 OR #6 OR #7 OR #8 OR #9 OR #10 OR #11 OR #12 OR #13 OR #14 OR # 15 OR #16 |  |
| 18 | Supervisors | preceptor | [Title/Abstract] |
| 19 |  | facilitator | [Title/Abstract] |
| 20 |  | supervisor | [Title/Abstract] |
| 21 |  | Faculty, medical | [MeSH Terms]^b^ |
| 22 |  | mentor | [MeSH Terms]^a^ |
| 23 |  | #17 OR #18 OR #19 OR #20 OR #21 OR #22 |  |
| 24 | Medical students | students, medical | [MeSH Term]^b^ |
| 25 |  | education, medical, undergraduate | [MeSH Term]^b^ |
| 26 |  | student* | [Title/Abstract] |
| 27 |  | undergraduate* | [Title/Abstract] |
| 28 |  | “Medical student*” | Title/Abstract |
| 29 |  | “Medical education” | Title/Abstract |
| 30 |  | #24 OR #25 OR #26 OR #27 OR #28 OR #29 |  |
| 31 |  | #23 AND #30 |  |
| 32 | Discipline of medicine | Medic* | [Title/Abstract] |
| 33 |  | Medicine | MeSH Term^b^ |
| 34 |  | #32 OR #33 |  |
| 35 |  | #31 AND #34 |  |
| 36 | First exposure | First | Title/Abstract |
| 37 |  | early | Title/Abstract |
| 38 |  | #36 OR #37 |  |
| 39 |  | #35 AND #38 |  |
| 40 | Preparedness | Clinical competence | MeSH term^a^ |
| 41 |  | Prepar* | Title/Abstract |
| 42 |  | Read* | Title/abstract |
| 43 |  | #40 OR #41 OR #42 |  |
| 44 |  | #39 AND #43 |  |

^a^ Search field Title/Abstract in non-MeSH term databases

^b^ Search term excluded from non-MeSH term databases
