# Supplementary material for: Perceptions of preparedness for the first medical clerkship: a systematic review and synthesis
Source: BMC Med Educ. 2016 Mar 12;16:89. doi: 10.1186/s12909-016-0615-3 (PMC4788861; doi:10.1186/s12909-016-0615-3)
Supplement: Additional file 3: Table S2. — Comprehensiveness of reporting—survey studies (DOCX 36 kb) [file 12909_2016_615_MOESM3_ESM.docx]

**Additional file 3: Table S2. Comprehensiveness of reporting – survey studies**

| **ITEM** | **Articles** | | |
| --- | --- | --- | --- |
|  | Shacklady *et al[20]* | Wenrich *et al[23]* | Prince *et al[21]* |
| **Participant Characteristics** |  |  |  |
| Sampling technique | ✓ | ✓ | ✓ |
| Exclusion criteria | ✓ | ✓ | ✓ |
| Response rate | ✓ | ✓ | ✓ |
| Characteristics of sample | ✓ | ✓ | ✓ |
| Characteristics of refusals |  | ✓ |  |
| **Survey administration** | | | |
| Method of approach or invitation | ✓ | ✓ | ✓ |
| Mode of delivery | ✓ | ✓ | ✓ |
| Incentive for survey completion |  | ✓ |  |
| Follow-up reminder |  | ✓ | ✓ |
| Anonymity |  | ✓ | ✓ |
| **Survey design** | | | |
| Piloting of survey tools and exercises |  |  |  |
| Description of survey development |  | ✓ | ✓ |
| Survey tools/ranking exercises provided or adequately described | ✓ | ✓ | ✓ |
| **Data analysis** | | | |
| Description of statistical analysis | ✓ | ✓ | ✓ |
| **Quotations (where applicable – Shacklady *et al[20]* and Prince *et al[21])*** | ✓ |  |  |
| **Qualitative analysis (where applicable – Shacklady *et al[20]* and Prince *et al[21])*** | ✓ |  |  |
