# Supplementary material for: Perceptions of preparedness for the first medical clerkship: a systematic review and synthesis
Source: BMC Med Educ. 2016 Mar 12;16:89. doi: 10.1186/s12909-016-0615-3 (PMC4788861; doi:10.1186/s12909-016-0615-3)
Supplement: Additional file 4: Table S3. — Characteristics of Included Studies (DOCX 45 kb) [file 12909_2016_615_MOESM4_ESM.docx]

**Additional file 4: Table S3. Characteristics of Included Studies**

|  | **Country of Origin** | **Aims of Study [study design]** | **Context** | | | **Findings** | **Interpretation of Findings** |
| --- | --- | --- | --- | --- | --- | --- | --- |
|  |  |  | **Setting / degree details** | **Design** | **Participants** |  |  |
| Shacklady (2009)[20] | UK | To quantify the effect of maturity on medical students’ transitions into the clinical environment and identify how experiences of transition might be improved  [matched cohort] | PBL-based,  5-year undergraduate program with 3 phases, with minimal clinical exposure in phase 1 | Cross-sectional web-based survey Survey focused on preparedness and consisted of 7 items (5 open-ended items, 2 Likert scale items) | 2 consecutive student cohorts in Year 3 (Phase 2):  Cohort: Mature aged students  N=29  Gender: 15M, 14F  Av Phase 1 performance^a^: 59.6 (range = 56-64)  Comparison: Non-mature aged students  N=58  Gender: 30M, 28F  Av Phase 1 performance^a^: 53.3 (range = 49-58)  RR: 42% | Mature aged students reported a   - Good transition (OR=6.1 p=0.002) - Drew on previous years in medical school (OR=2.7 p=0.04 - Drew on wide life experiences (OR=3.9 p=0.01)   Factors which were perceived to influence perceived transition included: familiarity with the clinical environment, relevance of clinical teaching, support during transition, preparatory experiences (work experience, learning and teaching knowledge, skills, early experience) | Mature aged students reported greater preparedness and a smoother transition into the clinical environment, than non-mature aged students.  The transition into the clinical environment was supported by   - Teacher support - Prior workplace experience - Clinically relevant knowledge - Confidence in skills |
| Wenrich (2010)[23] | USA | To determine teacher and learner expectations of clinical-skills training in preparation for clerkships  [cross-sectional, comparative] | Four-year graduate program; two years preclinical training followed by two years clinical clerkships. Pre-clinical training underpinned by Guided Bestide Learning pedagogy | Cross sectional web-based survey, which focused on the level of preparedness with respect to basic and advanced clinical skills and basic science knowledge (measured on a 5-point likert scale), when entering the clerkship | 1 student cohort, 3 months after commencing the clinical  N=115 (62%)  Clerkship faculty  N=56 (58%)  Gender: 38M, 18F  Pre-clinical faculty  N=30 (61%)  Gender: 15M, 15F  : | Preclinical faculty and students had higher expectations than clerkship faculty in all basic skills except for communication and teamwork (p<0.05)  Students had higher expectations in advanced skills than preclinical and clerkship faculty (p<0.05)  No significant difference in basic science knowledge expectations between the groups | Agreement is required between preclinical and clerkship faculty regarding expectations of preparedness of students prior to commencing the clerkship  Improved communication between students and faculty required to convey expectations of preparedness for the clinical clerkship |
| Prince (2005)[21] | Netherlands | To seek quantitative verification of the qualitative findings from the group’s previous focus group study[22] on students’ perceptions of the gap between pre-clinical and clinical training  [cross sectional] | PBL-based 6-year undergraduate program; four years theory-oriented preclinical training incorporating clinical skills training and two years clinical clerkships; PBL curriculum and | Cross-sectional mail-based survey consisting of 95 items (8 items on demographic characteristics, 77 items measured on a 5-point likert scale, 10 open-ended questions | Clerkship students  N=71 (67%)  Gender: 19M 52F  Mean age (SD): 23.1 (1.52) years | Student reported that the following impacted on their transition into the clinical clerkship; an increase in workload, variable patient contact, a change in learning expectations, positive and negative staff-student contact, lack of accessible knowledge, unfamiliar environment, uncertainty of their role and responsibilities | - More study time - Earlier patient contact to facilitate knowledge acquisition - Better introduction to the clerkship |
| Small (2008)[25] | USA | To identify the skills medical students perceive as essential and those skill aras students are most anxious about prior to starting clinical rotations  [cross-sectional] | Flexnarian-based 4-year graduate program; two years basic science non-PBL followed by two years clinical training; longitudinal clinical skills program during first two years including patient contact; 4-day clinical skills week prior to first clerkship | Paper-based survey consisted of 3 open-ended items | 2 student cohorts  Preclinical students (end of Year 2)  N=93 (76%)  Clinical students (9^th^ month of Year 3)  N=105 (90%) | Preclinical students”  Three most essential skills to be prepared for are history taking/ physical examination (73%), proficiency in oral case presentations (56%), generation of differential diagnosis (46%). Students were most anxious about oral presentations (30%)  Clinical students  Three most essential skills were interpersonal skills (80%), history taking/physical examination ((37%), time management (26%). Students were most anxious about time management and self care (40%) | Issues differ before and after students have made the transition. Preclinical curriculum should be aligned with student experiences during the clerkship |
| Prince (2000)[22] | Netherlands | To obtain a better insight into students’ experiences and opinions with regard to the transition from theory to practice  [focus group] | PBL-bassed, 6-year undergraduate program; four years theory-oriented preclinical training (including clinical skills training) and two years clinical clerkships; | Random selectedf fifth-year students for focus groups of 2-hour duration | Fifth-year students  N=20  Gender: 7M, 13F  Average time spent as clerks: 19.6 week (range 5-38) | Themes involved in the transition from preclinical to the clerkship include   - Transition - Contact with real patients - Knowledge - Practical skills - Learning | Narrow the gap between theory and practice3 by offering more practical experiences in the preclinical [phase and some problem-based tutorials in the clinical phase |
| Dornan and Bundy (2004)[27] | UK | To provide a rationale for integrating experience into early medical education (“early experience”)  [grounded theory analysis of semi-structured group interviews] | Three medical schools in the UK | semi-structured group interviews; staff and students attended separate sessions; grounded theory analysis0 | Purposeful sampling of 33 students from various years and 31 staff representing various curriculum aspects | The rationale for early experience would be to ease the transition; make students more confident to approach patients; motivate them; increase their awareness of themselves and others; contextualize their theoretical knowledge; teach intellectual skills; strengthen learning of behavioural and social sciences; teach them about the role of health professionals | Without early clinical experiences, the curriculum was socially isolating and divorced from clinical practice |
| O’Brien (2007)[24] | USA | To explore the relationship between students’ and clerkship directors’ perceptions of students’ struggles with the transition to clerkship.  [focus groups] | Ten US Medical Schools – details on curricula not provided | Data obtained from a large national study; responses gained from 16 interviews and 8 focus groups with clerkship directors and 11 focus groups with clerkship students | 83 clerkship students and 65 clerkship directors | Students reported difficulties understanding roles and responsibilities, adjusting to clinical cultures, performing clinical skills, learning the logistics of clinical settings, encountering frequent changes in staff, settings and content.  Clerkship directors identified students struggle with roles and responsibilities, performing clinical skills, adjusting to clinical cultures, difficulties applying knowledge to clinical reasoning and engaging in self-directed learning | Curricula and teaching strategies should be focused on areas which are not congruent between students and clerkship directors |
| Godefrooij (2010)[26] | Netherlands | To explore how students who have had preclinical patient contacts perceive the transition to clinical training and how they value these early patient contacts as preparation for learning in clinical practice  [focus group with data analysed using qualitative content analysis] | PBL-based 6--year undergraduate program; four years theory-oriented preclinical training with clinical skills training) and two years clinical clerkships; early contact with patients in Year 3 | Focus group interviews of 1.5 hours in duration, 2 focus groups were conducted with each of the 3 groups (for theoretical saturation) | Clerkship (Year 4) students who had completed 1 full week of their first rotation  N=21  Gender: 7M, 14F | Students reported increase in stress due to increased working hours and workload, uncertainty of what is expected of them and self-perceived lack of knowledge |  |
