# Supplementary material for: Perceptions of preparedness for the first medical clerkship: a systematic review and synthesis
Source: BMC Med Educ. 2016 Mar 12;16:89. doi: 10.1186/s12909-016-0615-3 (PMC4788861; doi:10.1186/s12909-016-0615-3)
Supplement: Additional file 5: Table S4. — Themes, theme descriptions and examples of coded text (DOCX 94 kb) [file 12909_2016_615_MOESM5_ESM.docx]

**Additional file 5: Table S4. Themes, theme descriptions and examples of coded text**

| **Theme** | **Definition** | **Quantitative Evidence** | **Examples** |
| --- | --- | --- | --- |
| Competence | Perceived deficiencies, need to acquire new skills | Preclinical faculty and medical students had significantly higher expectations than clerkship faculty for preparation in most basic clinical skills and students had higher expectations than all faculty for advanced clinical skills. ^14^  Knowledge was perceived to be sufficient by half the students for the behavioural science domain and by 25.4% and 18.5% for the domains of clinical science and basic science, respectively. The majority of students felt themselves to be well prepared and able to perform clinical duties.^15^ | There were qualitative differences between mature and non-mature students: Mature students expressed more confidence in their knowledge than non-mature students, though both would have liked more anatomy teaching.^16^  A lack of knowledge was the fifth most salient impression of clerkship students surveyed.^15^  Students report more deficiencies in knowledge than in clinical skills.^21^  Our students feel competent in taking histories and performing physical examinations, which we believe reflects the amount of curricular time devoted.^23^  What we are doing is a gentle introduction . . . [having] . . . a chat with patients . . . is something that is really difficult. Going and taking a history is one thing, but actually just going and having a chat with somebody is much more difficult. The history is quite a nice logical process, and you can learn that, and it is like rote learning really, but going and talking to somebody, making conversation with them, that is almost a first step.” ^25^  Students identified gaps or shortcomings in their pre-clerkship education as a primary source of their struggles, including knowledge that is decontextualized and not integrated with clinical knowledge used in practice, as well as deficient preparation in high-value practical skills such as documentation in real time.^22^  *"*I haven't even finished my second week as a clerk yet. I'm currently going through the absolute depression of ignorance." ^24^  “There's a lot you don't know yet. I find it very frustrating when I am asked a question and again and again I'm not able to give the correct answer”. ^24^ |
| Disconnection | Sense of disconnection between learning in the preclinical and clinical years |  | Students identified gaps or shortcomings in their pre-clerkship education as a primary source of their struggles, including knowledge that is decontextualized and not integrated with clinical knowledge used in practice.^22^  “I think the problem in the first two years is that we are only doing the psychology part, and not really applying it, so we become a bit ‘is this worth it at all?,’ whereas it does have its use, but . . . we don’t see it.” ^25^  Student learning in the clerkships appears to be driven by curiosity and exploration. They want to understand the patient problem. By contrast, they perceive their learning during the preclinical years as passive acquisition of knowledge, despite the problem-based system. ^21^  “The first two years . . . prepared me in terms of knowing the...theory of the diseases I now see but I had no idea.” ^16^ |
| Uncertainty | Confronting the uncertainty of clinical practice and applied medicine and what is expected of them as clerks | Over 57% of students reported that they were very uncertain at the beginning of their clerkship but the majority agreed that the uncertainty only lasted a few days^15^ | However, it was confusing for the students when different doctors had different notions of the correct way to perform a physical examination: "But you can never do it right, because each doctor has his own method and says: no, you have to do it this way. And next time, when you do it like that, another doctor will tell you: no, you should do it this way." ^24^  "I think what we mean here is this: you are put on a ward, which is challenging, but you don't know the rules. You don't know which person is nice and which person is not, and sometimes even how you should dress (...)." (CS2) ^24^  “Going into third year my preceptor gave me no expectations, no rules, nothing except for, “here we go, follow me.” You don’t know what his expectations are. Then you wonder, as far as being assessed, how is that going to work out?” ^22^  Students reported feeling insecure because they did not know what was expected of them.^21^  More than half the students wanted more information about what was expected of them as clerks.^15^ |
| Links to the future | Connection to the end result of being a doctor, the beginning of the development of a professional identity | Nearly 37% of students reported that the clerkship was their first experience of what it was like to be a doctor. ^15^ | "Finally it's beginning to get professional. Finally it's moving toward being a doctor." ^24^  “Having to get used to being in hospital, “wearing a white coat and being seen as a medic”. ^25^  “Hey, stand back for a moment, let’s see where this fits into the broader picture, what is it that doctors do, what are patients, what are populations, what is society?” ^25^  “I learn because I need the knowledge to help the patient”. ^21^  “You might learn the cut-off factor, which I understand . . . a lot of people think you’ve got to get as a doctor. You just go home and forget about things, but then maybe you’d not become as good a doctor if you can cut off that easily.” ^25^ |
| Part of the Team | Introduction to the healthcare team, roles, being part of the system, feeling like they belong, knowing where to stand, what to do and how to behave |  | “Experiencing the other health professions . . . in order . . . to get a feel for how they fit in with everything else.” ^25^  "What I find difficult to sense is, you know, when it is okay to speak up and when you had better keep quiet." ^24^  “I stopped being uncomfortable when I realized that my role on the team was to learn . . . from the situation and from the patients. So that allowed me to sort of ask more questions that I at first thought was beyond my boundaries to ask ‘cause it was personal information and to realize that we are doctors and if they’re going to tell anybody that they’re going to tell us.” ^22^  A more extensive introduction might have made matters easier, but even then, their prime concern in the first few days or weeks would have been with how to behave and act rather than with learning. ^15^  Issues causing anxieties for students were similar in both cohorts and included time management/self care, medical knowledge and the student role in the hierarchy system. ^23^  Both mature and non-mature students could feel superfluous: Mature: Fine although I still feel like a third wheel wherever I go”. ^16^  …transition from the preclinical phase to the clinical setting and its social implications. ^21^ |
| Time/ Workload | Workload, time spent on different tasks, time management | Nearly 75% of the students perceived a huge change in workload and thought they had insufficient time for studying.^15^ | "Yes, all that busyness. All at once your days are full. Now, during the winter period, I feel like some kind of caveman: you go to work in the dark and you come home in the dark. That really is a change for me." ^24^  “Nowadays I have a list of things I want to look up, so many I lack the time to study them all” ^21^  “There is a vast difference, whether you can loaf around all day, do your shopping, or whether you have to work all day, get home at 7 p.m. and have to study the whole evening”. ^21^  Neither clerkship directors nor students focused on overarching context factors such as duty hours regulations or the productivity demands of clinical teachers as factors. ^22^  Workload was the most salient early clerkship impression of clerkship students surveyed.^15^  Balancing their academic and work responsibilities with social and personal obligations apart from their training. ^23^  ..handling time constraints and travel commitments. ^16^ |
| Adjustment | Adjusting to the clinical environment, shock versus ease of transition, attitudes towards there being a period of adjustment | 62% of mature students described good transitions compared with 24% of non-mature students (*P*=0.002). ^16^  11.2% indicated that they had considered leaving medical school at the start of clerkship. They had difficulty adjusting to the daily routine and 40% said the transition was abrupt.^15^ | “They might experience as much excitement with less stress if they were “titred into it” and might “more or less understand the stress it might cause you, and [learn to] . . . deal with it better.” ^25^  “It is totally reversed, now I have to think from symptoms, whereas I used to study from diagnoses” ^21^  Students spoke of needing to build confidence to talk with patients and act appropriately in their presence “as medical students rather than friends”. ^25^  Mature students were more likely to feel positive about their transition into the clinical environment, and less likely to feel confused, daunted or overwhelmed...However, the clinical environment could be disconcertingly unfamiliar, even to them, and both mature and non-mature students valued peers’ and teachers’ support. ^16^  Acceptance:  “Eventually they’re going to struggle when they learn to apply it no matter where it is . . . there’s going to be a transition point that’s going to be rough for them.” ^22^  "If you do any other study there would also come a point where you'd have to start gaining work experience (...) and you start working, so it's just all in the game." ^24^  “I'm a clerk in order to learn, if I knew everything already then I wouldn't be here". ^24^  Shock:  Meeting seriously ill people challenged students. To be first insulated from it and then “dumped into a hospital environment . . . might be too much for you,” “scar you,” and teach you to “cut off”. ^25^  Being alert all the time and adapting to their new environment required considerable effort, not only mentally but also physically because of the long hours they had to work. ^21^  “The first week is so tiring, all these impressions, people who die, introducing yourself to everyone and always showing your best side”. (BC2) ^21^ |
| Curriculum | Actual and suggested educational strategies to increase preparedness for clerkship | 92.9% agreed that a good introduction would make the transition easier and 63.4% agreed that a general introduction should be provided to all new clerks. ^15^ | Furthermore, some of our students commented that it would help to include fourth year students as co-facilitators in the preclinical training courses to provide their insights based on firsthand experiences into the roles and expectations of students on the team. ^23^  Students identified gaps or shortcomings in their pre-clerkship education as a primary source of their struggles, including knowledge that is decontextualized and not integrated with clinical knowledge used in practice, as well as deficient preparation in high-value practical skills such as documentation in real time.^22^  “The first week there were so many things that I was expected to know right off the bat that I just didn’t feel comfortable with yet and a big thing was just getting oriented to the system, learning how documentation goes, how to read the electronic medical record, what different abbreviations mean, just how things run on the ward. So just some background things like that were difficult to get used to.” ^22^  They wanted to learn how to admit patients, particularly how to carry out a quick, structured, physical examination. ^15^  …suggested that preclinical experience in practice settings would have eased the transition. ^16^  A more gradual entry to the clinical environment, students suggested, would achieve a better balance of positives and negatives. ^25^  Contrary to expectations, PBL apparently fails to bring about the integration of basic and applied sciences, which it is supposed to enhance. ^21^  The students valued the introductory weeks of most rotations, which helped them refresh and integrate their prior knowledge and skills and fill the most important gaps in their knowledge, thereby improving their preparedness for the rotation. Nevertheless the students also noted a need for improvement in the educational quality of the introductory week in some rotations. "You are really stimulated to refresh your knowledge, for instance in cardiology and other subjects... That is really good." (CB2) "I don't think lectures are the ideal way to teach students for eight hours a day." ^24^ |
| Prior Life Experiences | Maturity, life experiences during or prior to medical school | 55% of mature students rated the statement ‘My previous years in medical school prepared me for year 3’ above the scale median compared with 31% non-mature students (*p=*0.04). 76% of mature students rated the statement ‘Experiences outside medical school prepared me for year 3’ above the scale median compared with 46% of non-mature students (*P*=0.01).^16^ | “Understanding other people’s lifestyles and value systems before moving to understanding those people, then, as patients.” ^25^  “In year 3, on our first time in the outpatient clinic we had prepared something but not everything. And then you feel: this patient has come here especially for you and you don't even know what to ask. And then you realise that you have a certain obligation towards patients, because they are so kind as to help you, you have to make sure you are well prepared." ^24^  Staff and students agreed that early experience could fill a gap, but their gaps differed. For staff, it was in students’ prior life experiences. Staff saw students as having generally limited prior life experience, “coming from a sheltered, protected school environment,” needing to encounter social diversity and develop social awareness, and needing time for intellectual and emotional development. ^25^  Mature [student]: I have a degree in a social science which . . . helps me appreciate the complexities surrounding an illness and the impact it has on peoples’ lives. I have worked in nursing homes and seen how the elderly and infirm are cared for on a day to day basis. ^16^  We conclude that mature students were significantly more likely to draw on the official undergraduate curriculum and their wider life experiences to have a smoother transition into the clinical environment. ^16^  “And it’s like “do it and no one’s going to hold you by your hand and drag you around the rest of your life.” You’re going to have to deal with it.” ^22^ |
| Learning | Learning styles, learning in a new environment, patients as tools for learning | ^15, 16^ | “It is very much easier to link what you have learnt if you can say: ‘Oh yes, well I saw somebody with that,’ and then that creates a picture in your mind, and it actually helps you to remember why things” ^25^  “One of the difficulties that faces a student going from the second to the third year is the change from learning from textbooks and resources provided within lectures . . . to actually learning from people . . . And, so some people can go running away back to their textbooks when you should really be learning how to learn from people themselves.” ^25^  “But what is difficult sometimes is that you have learned to first study a disease and then its symptoms, and now you have to reason the other way around and sometimes that's difficult." ^24^  “I do think the shift from what I term ‘bolus learning,’ you know, learn for the test next week and get to the next test—it’s very different from what they need to develop for their professional careers where it’s a lot more continuous learning, self directed learning, self reflection, recognizing one’s own gaps and filling those.” ^22^  “(. . . ) from learning we had to switch to applying our knowledge in practice” ^21^  However, it is clear that the distress of struggling interferes with optimal learning when students are unable to focus on patient-care tasks and are hesitant to ask questions or initiate action for fear of breaking rules or overstepping their boundaries. ^22^  “The transition between what, in first and second years, is a lot of lower-level learning objectives, memorization and what not, to trying to apply that to clinical situations. They still seem to try very hard to find out what the finite body of knowledge that they need to know is as opposed to kind of understanding that they need to apply that in new situations.” ^22^  “In the beginning I was occupied with what the physician wanted and with filling out papers, rather than with patients or learning” ^21^  Difficulties in the application of knowledge and skills. ^21^  Learning (more and faster in clinical practice) was the third most salient early clerkship impression. ^15^  Clinical learning could be ‘different’, ‘confusing’, ‘difficult’, ‘scary’ and ‘overwhelming’. ^16^ |
